# Effect of Replacing Lucerne Meal with *Seriphium plumosum* Meal in the Diet on Growth Performance, Methane Emission, In Vitro Rumen Fermentation Characteristics, and Rumen Microbial Community Composition of Nguni Steers

**DOI:** 10.3390/ani16213456

**Published:** 2026-11-02

**Authors:** Motswapo Esther Phoko, Jones Wilfred Ng’ambi, Tlou Julius Tjelele

**Affiliations:** 1 Range and Forage Science Department, Agricultural Research Council, Irene 0062, South Africa; 2 Department of Agricultural Economics and Animal Production, University of Limpopo, Polokwane 0727, South Africa; 3 Department of Agriculture and Animal Health, College of Agriculture & Environmental Sciences, University of South Africa, Roodepoort 1709, South Africa; ngambjw@unisa.ac.za (J.W.N.); tjeletj@unisa.ac.za (T.J.T.)

**Keywords:** cattle, condensed tannins, enteric methane, rumen bacteria, woody plant species

## Abstract

This study assessed the effects of replacing lucerne meal with *Seriphium (S.) plumosum* meal on growth performance, methane emissions, rumen fermentation characteristics, and rumen bacterial community composition of Nguni steers. Twenty-eight 22-month-old Nguni steers (average body weight 300 ± 10 kg) were randomly assigned to diets containing 0%, 10%, 20%, or 30% of *S. plumosum* meal in a completely randomized design. The diets were formulated to be isocaloric to meet the nutrient requirements of growing Nguni steers. Feed intake, nutrient digestibility, and live weight gain were measured over a 90-day feeding period. Methane emissions were recorded using a laser methane detector, while rumen fermentation characteristics were evaluated in vitro. Rumen microbial communities were characterized using 16S rRNA gene sequencing. Increasing *S. plumosum* inclusion had no effect (*p* > 0.05) on dry matter intake, nutrient digestibility, live weight gain, or methane emissions, indicating that replacement of lucerne meal did not compromise production performance. However, dietary treatments influenced (*p* < 0.05) gas production throughout the incubation period (3–48 h). The 10% diet (T2) produced the highest gas volumes, with values declining at 20% (T3) and 30% (T4). Cumulative gas production after 48 h followed a similar pattern. Significant linear, quadratic, and cubic responses (*p* < 0.01) indicated a non-linear response of gas production to increasing dietary inclusion of *S. plumosum*. In vitro organic matter digestibility, ammonia–nitrogen concentration, volatile fatty acid profiles, and the acetate-to-propionate ratio, demonstrated changes (*p* < 0.05) in rumen fermentation dynamics. Heatmap analysis of the rumen microbiota revealed clear shifts in bacterial community composition with increasing *S. plumosum* inclusion in the diet. Therefore, *S. plumosum* meal has potential as a locally available alternative forage resource for partial replacement of lucerne meal in Nguni steer diets.

## Simple Summary

Replacing lucerne meal with *Seriphium plumosum* meal in the diet of Nguni steers has the potential to modify rumen microbial community composition without negatively affecting animal performance. In this study, lucerne meal was replaced with *S. plumosum* meal at 0%, 10%, 20%, and 30% inclusion levels in diet, and the effects on growth performance, methane emissions, rumen fermentation characteristics, and microbial communities were evaluated. Animal performance and methane intensity were not affected by dietary treatment. However, significant differences were observed in gas production throughout the incubation period (3–48 h) and in vitro organic matter digestibility, indicating changes in rumen fermentation dynamics. Heatmap analysis indicated that the bacterial community exhibited genus-specific responses to dietary inclusion of *S. plumosum*, although no pronounced shifts in the dominant bacterial populations were evident across treatments. Findings in this study indicated that *S. plumosum* can moderately replace lucerne meal as an alternative forage supplement without compromising cattle productivity while promoting changes in rumen microbial ecology.

## 1. Introduction

Livestock production in South Africa depends extensively on natural rangelands, where the quality and quantity of natural pasture deteriorate during dry seasons, this situation is exacerbated by climate change [1]. Climate change directly reduces both the quantity and quality of available feed resources, thereby adversely affecting livestock production [1,2]. Therefore, to address feed shortage and poor-quality feed in dry season, indigenous woody plants are increasingly considered as alternative supplementary feed resources for ruminants. Woody plants contain fermentable carbohydrates and secondary metabolites, amongst others; condensed tannins (CT), phenolic compounds, oils, flavonoids, etc., which have shown to improve nutrient availability and reduce methane (CH_4_) production by ruminants [3,4]. Livestock contributes about 32% of anthropogenic methane emissions with cattle accounting for about 77% of the total enteric methane emissions [5,6]. During ruminal fermentation, hydrogen (H_2_) and carbon dioxide (CO_2_) produced by fermentative microorganisms are used by methanogenic archaea, through the hydrogenotrophic pathway, to produce enteric CH_4_, thereby maintaining a low partial pressure of hydrogen that favours efficient microbial fermentation [7]. Therefore, methanogens are the main role players in producing enteric CH_4_ emissions in ruminants, and that makes them the key targets for enteric CH_4_ production mitigation reduction strategies [8]. Enteric methane emission results in a loss of 2–12% gross dietary energy, leading to reduced feed efficiency utilization by ruminant and consequently a decline in ruminant performance [9].

Numerous studies have reported the potential of tannin-rich woody plants as alternative feed resources for ruminants, owing to their potential to improve nitrogen utilization, modulate rumen fermentation, and mitigate CH_4_ emissions [10]. *Seriphium plumosum* is an indigenous South African woody plant species that is increasingly invading grassland ecosystems and adversely affecting natural pasture availability [11]. Although *S. plumosum* has been studied for its botanical characteristics, phytochemical constituents, ecology and biological activities [11,12], limited research has investigated its potential as a feed ingredient in ruminant diets, particularly its effects on animal performance, nutrient utilization, enteric methane emissions, rumen fermentation and rumen microbial populations. *Seriphium plumosum* contains bioactive secondary metabolites, particularly condensed tannins and phenolic compounds, which may influence ruminal microbial activity and fermentation processes and potentially affect CH_4_ production and animal performance [10,12,13]. Furthermore, *S. plumosum* contains a moderate crude protein (CP) concentration (7–12%), which may contribute to ruminant nutrient supply during the dry season when the nutritional quality of grasses is low [12]. Generally, these characteristics highlight the potential of *S. plumosum* as a supplementary feed resource for ruminants. Limited information is available about its effects on growth performance, nutrient digestibility, rumen fermentation, methane emissions, and rumen microbial populations; this makes the plant more fundamental for inclusion in cattle diets.

Lucerne meal was selected as the ingredient to be replaced because it is a conventional, high-quality forage widely used as a protein and fibre source in beef cattle diets [14]. *Seriphium plumosum* was selected for use as feed ingredient in this study because of its high abundance and its significant contribution to bush encroachment and consequently reduction in grazing capacity. Although *S. plumosum* differs nutritionally from lucerne, with higher concentrations of structural carbohydrates (NDF = 66.1% vs. 38–45% DM; ADF = 37.0% vs. 28–35% DM), its CP concentration may still contribute to meeting ruminant nutrient requirements when incorporated into nutritionally balanced diets [12,14,15]. It was therefore hypothesized that replacing the lucerne meal with *S. plumosum* meal will enhance the production performance of Nguni steers, maintain rumen microbial ecosystem and reduce CH_4_ emission to demonstrate its potential as a sustainable and locally available resource feed for cattle. This study aimed at evaluating the effect of replacing lucerne hay with dietary *S. plumosum* meal inclusion levels in diet on growth performance, methane emissions, in vitro rumen fermentation characteristics, and rumen microbial community composition of Nguni steers.

## 2. Materials and Methods

### 2.1. Study Area, Animal Management, Treatments and Experimental Design

The study was conducted at the feedlot facility of the Agricultural Research Council–Animal Production (ARC-AP), Gauteng Province, South Africa, during the summer months of 2020. The facility is located at 25°53’ S and 28°11’ E, with mean ambient temperature of 15.8 °C in winter and 28 °C in summer (https://en.climate-data.org). The study area has a predominantly dry winter season from April to September, with most rainfall occurring during the summer months from October to March; the mean annual rainfall at the Irene weather station is approximately 696 mm [16].

*Seriphium plumosum* woody plant was harvested at a private farm in Vanderbijlpark, Gauteng Province, South Africa, between January and March 2020. Edible parts (leaves and branches) were harvested using forage harvester, sun-dried for five days, baled, and milled through a 2 cm sieve. The milled material was bagged and stored in a well-ventilated, dry, shaded area until commencement of feeding trial.

Twenty-eight Nguni steers aged 22 months old; weighing 300 ± 10 kg initial body weight were randomly assigned to four dietary treatments containing 0% (T1_0_), 10% (T2_10_), 20% (T3_20_), or 30% (T4_30_) of *S. plumosum* meal in a completely randomized design, with seven steers replicated per treatment. Random allocation of steers into singles pens (12 m^2^ _=_ 3 m × 4 m) was done to ensure that each steer has an equal access to diets. The selection of seven steers sample size per treatment was selected based on comparable nutritional studies evaluating browse species in ruminants, where treatment groups of six to eight animals were recommended to detect biological production performance. The nutrients requirements for steers were (dry matter 6.5–8 kg DM/day, crude protein 11–13% DM, neutral detergent fiber 30–24% DM, acidic detergent fibre 18–25% DM). Steers underwent a 21-day adaptation period to their respective diets and were trained to feed from individual feeding troughs prior to the 90-day actual feeding trial. All steers were individually fed and provided with ad libitum access to clean water and feed. Each steer represented the experimental unit, and they were all managed under similar environmental set up throughout the experiment. The experimental feed ingredients proportions and chemical composition of diets are presented in Table 1.

### 2.2. Chemical Analyses

Feed samples from each set of the experimental diets were analyzed separately, and the analytical values were averaged for each treatment. Diet samples were oven-dried at 60 °C for 72 h and subsequently ground prior to chemical analysis. Dry matter (DM) content was determined according to AOAC Official Method [17]. Dried samples were ground to pass through a 1 mm sieve (Wiley Mill, Standard Model 3, Arthur H. Thomas Co., Philadelphia, PA, USA) and stored in labelled airtight containers for further analysis. Crude protein (CP) was determined by the Kjeldahl method according to AOAC Official Method 984.13 and was calculated as total nitrogen × 6.25 [18]. Ether extract (EE) was determined by Soxhlet extraction according to AOAC Official Method 920.39, while acid detergent fiber (ADF) was determined according to AOAC Official Method 973.18 [17]. Neutral detergent fiber (NDF) was determined according to the method described by Van Soest et al. [18]. The metabolizable energy (ME) content of the experimental diets was estimated from the chemical composition of the feed ingredients using the predictive equations described by the National Research Council (NRC) [19]. The crude fiber (CF), calcium (Ca), phosphorus (P), and crude protein (CP) contents of the experimental diets and *S. plumosum* meal were determined according to the methods described by McDonald et al. [20]. Condensed tannin (CT) content of *S. plumosum* and the experimental diets were quantified according to the method described by Hattas and Julkunen-Tiitto [21].

### 2.3. Feed Intake, Live Weight Gain, Digestibility

Daily feed intake was calculated as the amount of feed offered minus the weight of feed refusals collected each day [20]. To determine live weight gain, steers were weighed fortnightly using a Micro T7E heavy-duty cattle weighing scale (Scalerite (Pty) Ltd, Johannesburg, South Africa) Johannesburg, South Africa. Daily live weight gain was calculated as the difference between the final and initial body weight divided by the number of feeding days. A digestibility trial was conducted using the total fecal collection method to determine the apparent nutrient digestibility, following the procedure described by Schneider & Flatt [22]. Fecal output was composed from each steer over five consecutive days (i.e., from day 85–89) of the experimental period from concrete single pens floors. Collected feces were thoroughly mixed, weighed, and approximately 10% of the daily output was pooled as a representative subsample [22]. Fecal subsamples were stored at −20 °C until conclusion of the collection period, after which they were oven-dried at 60 °C and ground to pass through a 1 mm screen. The analysis of dry matter (DM), organic matter (OM), crude protein (CP), neutral detergent fibre (NDF), and acid detergent fibre (ADF) were conducted using the standard procedure by Van Soest et al. [18] and AOAC methods [17]. 

Apparent nutrient digestibility coefficient was calculated as:

Digestibility (%) = Nutrient intake − Fecal nutrient output × 100

where nutrient intake and fecal nutrient output were expressed on a DM basis, as described by Schneider et al. [22]. Individual steer served as the experimental unit for all digestibility measurements. 

### 2.4. Measurements of CH_4_ Emission Using a Laser Methane Detector

Daily enteric CH_4_ emissions were measured for 10 consecutive days during the feeding period (i.e., from day 80–89) from individually penned steers using a laser methane detector (LMD) (Crowcon Detection Instruments Ltd., Tokyo, Japan). Methane emission measurements were taken by directing the LMD to the auxiliary of each steer from approximately 1 m distance to be able to detect CH_4_ emission without disturbing animal activity [23,24]. Measurements were repeated five times per session with 1-min intervals to capture different stages of the respiratory cycle [23]. Methane emissions were measured 2 h after the morning feeding to accord with the period of rumination, when enteric CH_4_ production is preeminent [23]. Methane emissions were manually recorded, and the average of thirty repeated measurements reflected the daily CH_4_ emission per steer. The averaged value of CH_4_ output from induvial steer were expressed as parts per million metres per day (ppm-m/d) [23]. 

### 2.5. In Vitro Gas Production, In Vitro Organic Matter Digestibility, Ammonia-Nitrogen and Volatile Fatty Acid as Influenced by S. plumosum Meal

To determine gas production, approximately 900 mL of rumen fluid was collected from each donor steer before morning feeding. The donor animals were healthy matured Nguni steers fed a basal diet consisting of lucerne hay and grass silage, which was offered twice daily, with ad libitum access to clean drinking water. The collected ruminal fluid was immediately filtered through four layers of cheesecloth into a pre-warmed thermos flask, then transferred into pre-warmed insulated flasks flushed with carbon dioxide (CO_2_) and transported to the laboratory for use as the inoculum in the in vitro fermentation analysis [24,25]. A buffer mineral solution was prepared according to standard procedures by Menke and Steingass [26]. Prior to incubation, 200 mg of dried diet substrates containing 0%, 10%, 20%, or 30% of *S. plumosum* meal were weighed into 120 mL serum bottles following the method of Theodorou et al. [25]. *Monensin* (Rumensin^®^; Elanco, Johannesburg, Gauteng, South Africa) was included as a positive control at a dose of 15 mg/kg DM feed, based on the manufacturer’s recommendation, and as previously described by Akanmu et al. [27]. Three blank bottles were included in each incubation run. Gas pressure from each serum bottle was incubated according to the pressure transducer technique of Tilley and Terry [28] and measured at 3, 6, 12, 24, and 48 h using a pressure transducer connected to a digital data tracker (Tracker 220 series indicators; Omega Engineering Inc., Swedesboro, NJ, USA). Gas pressure readings were recorded in psi and subsequently converted to gas volume. Gas volumes were corrected to a dry matter basis and expressed as mL/g DM. Gas production volumes measured at different time intervals were summed to estimate cumulative gas production over 48 h [25]. Incubation was terminated at 48 h by placing the bottles on ice. The samples were then centrifuged at 4500× *g* for 15 min, and 5 mL of the supernatant was collected and stored at −20 °C for in vitro organic matter digestibility (IVOMD) and volatile fatty acid (VFA) analysis [28,29].

In vitro organic matter digestibility (IVOMD) was determined after 48 h on the incubation deposit using the two-stage digestion procedure described by Erwin et al. [29] and modified by Engels and Van der Merwe [30]. Briefly, the undigested residue was recovered, and IVOMD was calculated from the difference between the initial organic matter and the organic matter remaining after fermentation [28,30].

Fermentation fluid was centrifuged at 10,000× *g* for 15 min, and the supernatant was mixed with 25% metaphosphoric acid, stored at −20 °C, and analyzed for VFA [29]. Volatile fatty acid (VFA) concentrations (acetate, propionate, butyrate, iso-butyrate, valerate, and iso-valerate) were determined by gas chromatography equipped with a flame ionization detector (GC-FID) then deproteinization of fermentation fluid was conducted with metaphosphoric acid, following the method of Erwin et al. [29].

### 2.6. Deoxyribonucleic Acid (DNA) Extraction and 16S Ribosomal Ribonucleic Acid (16S rRNA) Sequencing Analysis for Bacterial Population Profiles in Rumen Fluid

Rumen fluid samples were obtained from experimental animals at the end of the feeding trial before the morning feeding using a flexible oral stomach tube connected to a vacuum pump. To minimize saliva contamination, the initial 100–200 mL of ruminal fluid was rejected before the actual 50 mL of ruminal fluid was collected. Samples evidently contaminated with saliva or feed fragments were recalled and recollected. Rumen fluid was filtered through four layers of sterile cheesecloth to remove large feed particles, then added to 50 mL sterile polypropylene tubes, immediately placed on ice, and transported to the laboratory. Samples were then stored at −80 °C until microbial DNA extraction to preserve nucleic acid integrity [31]. Genomic DNA was extracted from individual rumen fluid samples, and transferred into sterile cryovials, immediately placed on ice, and stored at −80 °C until genomic DNA extraction for 16S rRNA gene sequencing [32].

The V3–V4 hypervariable region of the bacterial 16S rRNA gene was amplified using universal primers 338F (5′-ACTCCTRCGGGAGGCAGCAG-3′) and 806R (5′-GGACTACCVGGGTATCTAAT-3′), which have been widely applied for bacterial community profiling using Illumina sequencing platforms [33]. Bacterial DNA was extracted from rumen fluid samples collected from all 28 Nguni steers in triplicate using 300 μL of sample per replicate with the FastDNA™ Spin Kit for Soil (MP Biomedicals, Santa Ana, CA, USA) according to the manufacturer’s instructions, following procedures commonly used for rumen microbial DNA extraction [33]. Rumen fluid samples were collected and processed individually from each steer. No composite samples were prepared; each steer was treated as an independent biological replicate for microbial DNA extraction and sequencing. A total of 84 sterile cryovials (three aliquots per steer) were used to collect and preserve rumen fluid samples for microbial DNA extraction. One vial was used for DNA extraction, while the second and third served as a backup sample and was stored at −80 °C. An additional purification step was performed to remove potential PCR inhibitors. Samples were washed and resuspended in a humic acid wash solution containing sodium phosphate buffer, MT buffer (provided with the FastDNA™ Spin Kit for Soil), and 5.5 M guanidine thiocyanate according to the manufacturer’s protocol (MP Biomedicals, 2018). Following settling of the binding matrix, the supernatant was transferred to SPIN™ filters, and DNA was purified by centrifugation. Deoxyribonucleic acid was eluted in 50 μL of DNase/Pyrogen-Free Water supplied with the kit (MP Biomedicals, 2018). Deoxyribonucleic acid concentration was quantified using a Qubit™ Fluorometer (Invitrogen, Thermo Fisher Scientific, Waltham, MA, USA) with the Qubit™ dsDNA High Sensitivity Assay Kit, yielding DNA concentrations ranging from 45.7 to 148 ng/μL (Thermo Fisher Scientific, 2015) [34]. 

PCR amplification of the V3–V4 regions of the bacterial 16S rRNA gene was performed using universal primers 338F (5′-ACTCCTAC GGGAGGCAGCAG-3′) and 806R (5′-GGACTACHVGGGTWTCTAAT-3′) as previously described by Zhou et al. [33]. Sequence analysis was performed using the QIIME pipeline as previously described by Caporaso et al. [35]. Sequence reads were clustered into operational taxonomic units (OTUs) at 97% sequence similarity using an open-reference OTU-picking strategy. The most abundant sequence within each OTU was selected as the typical sequence and aligned against the Greengenes core reference database using PyNAST [35,36]. Taxonomic classification of OTUs was executed using the Ribosomal Database Project (RDP) classifier with a minimum confidence threshold of 80% [37]. Operational taxonomic unit tables were subsampled according to the sample with the lowest sequencing depth to equalize sampling effort [35]. For further univariate analysis, OTUs containing more than 5786 reads (0.1% of total reads) were reserved. One sampling period for one steer was removed during QIIME (1.9.1) processing due to comparatively low read counts in all three replicates (0.05% of total reads compared with other samples) [35].

A total of 378 bacterial genera were identified through 16S rRNA gene sequencing. For this study, the statistical analysis and visualization of ten genera were selected based on their mean relative abundance across all rumen samples (≥1%), ecological relevance in rumen fermentation, and responsiveness to dietary *S. plumosum* meal inclusion in diet. Although rumen fluid was collected from all 28 steers, due to sequencing costs only four representative samples per treatment (*n* = 4) were selected for 16S rRNA gene sequencing. The selection of four samples per treatment per genera was regarded as representative of the treatment mean. Focusing on the dominant bacterial genera enables biological interpretation by accenting the taxa that contribute most considerably to rumen fermentation and nutrient metabolism while reducing the influence of low-abundance taxa that may contribute limited functional information.

### 2.7. Statistical Analysis

All data was analyzed using one-way analysis of variance (ANOVA) in SAS version 9.4 (SAS Institute Inc., Cary, NC, USA) [38] to determine the effect of dietary *S. plumosum* inclusion levels on feed intake, live weight gain, digestibility, methane emission, in vitro rumen fermentation parameters and rumen microbial composition. The statistical model used was:

*Yi, j* = *μ* + *T_i_* + *e _i j_* Y_{*_ij_*} = \mu + *T_i_* + e_{*_ij_*} *Y_ij_* = μ + *T_i_* + *e _ij_*

where *Y_ij_* *Y_*{*_ij_*} *Y_ij_* is the observed response variables (feed intake, live weight gain, digestibility, methane emission, in vitro rumen fermentation parameters and rumen microbial composition), μ\muμ is the overall mean (mean for each variable across the diets), *T_i_ T_i_ T_i_* is the fixed effect of dietary treatment (0, 10, 20, and 30% *S. plumosum* inclusion), and *e_ij_ e*_{_ij_} *e_ij_* is the random residual error (accounts for the variation among individual steers that is not explained by the dietary treatments). When treatment effects were significant (*p* < 0.05), means were separated using Tukey’s Honest Significant Difference (HSD) test [39], while orthogonal polynomial contrasts were used to evaluate linear, quadratic, and cubic responses to increasing dietary *S. plumosum* inclusion. Orthogonal polynomial contrasts were performed because dietary *S. plumosum* inclusion levels (0, 10, 20, and 30%) signified similar spread-out quantitative treatments. These contrasts were used to determine whether the response variables changed linearly or showed quadratic or cubic trends as dietary of *S. plumosum* inclusion increased. The study used a completely randomized design with dietary treatment specified as a fixed effect and steers as experimental unit. No additional random blocking factors were included because animals were randomly allocated to treatments.

## 3. Results

### 3.1. Effect of Replacing Lucerne Hay with S. plumosum Meal in Diet on Nutrient Intake, Nutrient Digestibility, Live Weight Gain and Methane Intensity of Nguni Steers

As shown in Table 2, dietary inclusion of *S. plumosum* had no significant effect (*p* > 0.05) on DM intake, CP intake, NDF intake, ME intake, DM digestibility, CP digestibility, NDF digestibility, ME digestibility, initial body weight, final body weight, body weight gain, average daily gain, or methane emissions *(p* = 0.62). Although significant linear and quadratic polynomial trends were revealed for CP intake, NDF intake, and NDF digestibility, these trends were not accompanied by significant overall treatment effects Table 2 (*p* = 0.62).

### 3.2. In Vitro Gas Production, In Vitro Organic Matter Digestibility, Ammonia-Nitrogen and In Vitro Volatile Fatty Acid Profiles) as Influenced by Dietary S. plumosum Meal

The effects of replacing lucerne meal with increasing levels of *S. plumosum* (0, 10, 20, and 30%) meal on in vitro rumen fermentation characteristics are presented in Table 3. Gas production was significantly (*p* < 0.01) influenced by *S. plumosum* inclusion in diet at all incubation periods (3, 6, 12, 24, and 48 h). Gas production increased markedly from the control diet (T1; 28.81 mL/g DM) to diets containing *S. plumosum* meal, with the highest cumulative gas production observed at 10% inclusion (T2; 77.09 mL/g DM), followed by 20% (T3; 70.36 mL/g DM) and 30% (T4; 61.29 mL/g DM). Cumulative gas production after 48 h was significantly affected by *S. plumosum* inclusion in diet (*p* < 0.01). The orthogonal polynomial contrasts showed significant linear, quadratic, and cubic responses (*p* < 0.01), indicating that the relationship between *S. plumosum* inclusion level and cumulative gas production was not simply proportional. Orthogonal polynomial contrasts demonstrated significant (*p* < 0.01) linear, quadratic, and cubic responses in gas production variables with increasing levels of *S. plumosum* meal inclusion in diet. In vitro organic matter digestibility (IVOMD) was significantly affected by *S. plumosum* inclusion in diet (*p* < 0.01). Digestibility increased significantly from 48.93% in the control diet (T1; 0%) to 75.32% at 10% *S. plumosum* inclusion (T2), followed by a progressive decline at higher inclusion levels (67.62% at 20% inclusion and 61.36% at 30% inclusion).

The highest IVOMD value observed at 10% inclusion indicates that moderate inclusion of *S. plumosum* enhanced organic matter degradation compared with control diet. Ammonia-Nitrogen concentration was affected by dietary inclusion of *S. plumosum* (*p* = 0.021). The lowest NH_3_-N concentration occurred at 10% inclusion (31.69 mg NH_3_-N/100 mL), while the control and 30% inclusion diets showed the highest values (33.68 and 33.67 mg NH_3_-N/100 mL, respectively). Significant linear (*p* = 0.012) and quadratic (*p* = 0.044) responses showed a non-linear response of NH_3_-N to increasing *S. plumosum* inclusion, whereas the cubic response was not significant (*p* = 0.318).

The inclusion levels of dietary *S. plumosum* significantly (*p* < 0.01) affected total volatile fatty acid (TVFA) concentration and individual VFA profiles excluding Iso-valeric acid (Table 3). The VFA profile indicated that replacing lucerne meal with *S. plumosum* significantly altered rumen fermentation (*p* < 0.01). However, the response was not consistently linear. Most VFAs showed significant quadratic and cubic responses, indicating that the greatest changes occurred at the transitional inclusion level (20%) rather than gradually with increasing inclusion. Acetic acid concentration was significantly affected by dietary inclusion of *S. plumosum* meal (*p* < 0.01).

The significant linear response indicated that, overall, increasing *S. plumosum* inclusion influenced cumulative gas production across treatments. However, the response pattern was better explained by the significant quadratic and cubic effects, because gas production increased abruptly at 10% inclusion and then declined at higher inclusion levels. This suggests an optimum inclusion level of 10% *S. plumosum* meal for exciting microbial fermentation and gas production, whereas further inclusion (20–30%) reduced the response. Propionic acid concentration was significantly reduced with increasing *S. plumosum* inclusion (*p* < 0.01). A similar pattern was observed with linear, quadratic, and cubic responses (*p* < 0.01).

### 3.3. Rumen Microbial Composition

Figure 1 illustrates the mean relative abundance of the ten selected bacterial genera in the rumen fluid of Nguni steers fed diets containing 0, 10, 20 and 30% *Seriphium plumosum* meal. The selected genera were identified from a total of 378 detected bacterial genera based on their total relative abundance across all rumen samples, ecological relevance, and apparent responsiveness to dietary treatments. Descriptively, *Erysipelotrichaceae* UCG-009 showed the greatest mean relative abundance in steers fed the control diet (2.70%) and was less abundant in the *S. plumosum* diets (0.21–0.46%). *Lachnobacterium* showed relatively high abundances in 0% (2.47%) and 20% (2.34%) treatments, whereas *Ligilactobacillus* was most abundant in the 20% treatment (1.70%). *Ignavigranum* reached its highest mean abundance in the 10% *S. plumosum* treatment (0.91%), while *Kocuria* was most abundant in the 30% treatment (0.71%). The remaining selected genera occurred at relatively low mean abundances (<0.7%) across treatments.

## 4. Discussion

### 4.1. Effect of Replacing Lucerne Hay with S. plumosum Meal in Diet on Nutrient Intake, Nutrient Digestibility, Live Weight Gain and Methane Intensity of Nguni Steers

Replacement of lucerne meal with *S. plumosum* meal did not affect feed intake, nutrient digestibility, growth performance, or enteric methane emission of Nguni steers. These findings suggest that the inclusion of *S. plumosum* in diet did not negatively affect diet acceptance by steers. This is important because some woody browse species containing secondary metabolites, particularly CT, may impair feed intake when present at higher concentrations due to astringency effects and reduced feed preference [40]. Similar findings have been reported for *Leucaena leucocephala*, where inclusion of the tannin-containing forage did not adversely affect feed intake and was associated with improved nitrogen utilization [41]. The CT concentrations in the present study were relatively low (0.021–0.032% DM), which may explain the absence of adverse effects on feed intake, as these concentrations were likely insufficient to impair palatability or nutrient utilization [42].

Despite variation in dietary CP intake in the current study, feed intake, growth performance, and nutrient digestibility remained unchanged. These findings suggest that the additional dietary protein did not improve nutrient utilization by the steers. Moreover, the dietary protein supplied was likely sufficient to meet the requirements of the growing Nguni steers, such that increasing CP beyond their requirements conferred no additional benefit [19]. *Seriphium plumosum* used in this study contained 2.3% CT, which may facilitate the formation of reversible complexes with dietary proteins, thereby reducing ruminal protein degradation and altering nitrogen availability to rumen microorganisms [10].

Similar DM digestibility and CP digestibility observed across treatments suggest that replacing lucerne with *S. plumosum* did not compromise nutrient availability to steers. Condensed tannins may influence protein utilization by forming reversible complexes with dietary proteins, reducing excessive ruminal protein degradation while allowing protein release in the lower digestive tract [43]. Therefore, the stability of nutrient digestibility observed in this study indicate that the CT in *S. plumosum* provided a protective effect without causing inhibition of microbes [44]. Similar responses have been observed in ruminants consuming moderate levels of CT (20–45 g CT/kg DM) forage diets, where nutrient digestibility remained unaffected or improved due to enhanced nitrogen efficiency [42].

Although increasing *S. plumosum* inclusion increased dietary fibre intake, the absence of differences in growth performance indicates that the increased fibre did not restrict nutrient availability sufficiently to impair animal productivity [45]. Similar observations were reported by Choongo et al. [46] who found that supplementation with *Dichrostachys cinerea* significantly increased daily live weight gain in Friesian bulls during the dry season. Similarly, Ravhuhali et al. [47] reported that several indigenous South African woody browse species, including *Vachellia karoo*, *Dichrostachys cinerea*, *Grewia occidentalis*, *Ziziphus mucronata*, and *Senegalia nigrescens*, have potential as supplementary feed resources despite their relatively high fiber concentrations. These findings suggest that an increase in dietary fiber does not necessarily impair animal performance when nutrient availability and fiber digestibility are maintained [48]. For example, [19] reported that diets containing approximately 25–35% NDF (DM basis) generally support optimum rumen function and feed intake in cattle, [19] recommended dietary NDF concentrations of approximately 28–34% DM for growing beef cattle to maintain rumen health and productivity. Despite the high fibre content of *S. plumosum* meal (NDF = 66.1%; ADF = 37.0%), steers in the present study maintained growth performance, suggesting that the increased fibre fraction did not affect nutrient utilization. This response may be attributed to the ability of rumen microorganisms to degrade fibrous components when adequate nitrogen availability and fermentable energy are supplied [49,50]. Similar average daily gain among treatments further demonstrates that *S. plumosum* can replace part of the lucerne component without reducing growth potential in Nguni steers.

Previous studies have shown that indigenous browse species can support animal performance when diets provide adequate CP and energy to meet the nutrient requirements of growing cattle, even when structural carbohydrate concentrations are relatively high [19,51]. For instance, the high fibre concentration of the experimental diets in the current study showed that the inclusion of *S. plumosum*, in diets was characterized by a high structural carbohydrate content. Dietary fibre is essential for maintaining normal rumen function, stimulating chewing activity, and promoting saliva production, which helps stabilize ruminal pH [50]. Furthermore, Nguni cattle are well adapted to diverse production systems and can utilize fibrous, relatively low-quality forage resources [51]. Therefore, the fibre concentrations used in the present study were considered suitable for evaluating *S. plumosum* as a locally available forage resource for Nguni steers.

Although dietary CT concentrations increased with *S. plumosum* inclusion (0.021–0.032% DM), CH_4_ emissions were unaffected, indicating that these low CT concentrations were insufficient to influence ruminal methanogenesis [13]. The current findings are consistent with previous reports that significant CH_4_ mitigation generally requires higher concentrations and appropriate tannin structures [13,42]. Condensed tannins may reduce CH_4_ production through direct reserve of methanogens or indirectly by reducing hydrogen availability through altered fermentation pathways [8]. However, CH_4_ mitigation responses to tannin-containing diets are highly variable and depend on CT concentration, chemical structure, forage maturity, and adaptation period [13,52]. Previous studies have shown that the effects of low to moderate CT concentrations on enteric CH_4_ production are variable, with methane responses depending on CT concentration, chemical structure, and their effects on ruminal fermentation [44,53]. Therefore, the absence of CH_4_ reduction in the current study does not necessarily indicate a lack of biological activity of the diets but rather suggests that *S. plumosum* inclusion at the levels evaluated influenced ruminal fermentation characteristics and bacterial populations without substantially suppressing methane formation [54].

### 4.2. In Vitro Gas Production, In Vitro Organic Matter Digestibility, Ammonia-Nitrogen (mg/dL) and Volatile Fatty Acid (VFA) Profile as Affected by Dietary Replacement of Lucerne Hay with S. plumosum Meal in Diets

The 10% replacement of lucerne hay with *S. plumosum* influenced ruminal fermentation characteristics in a dose-dependent manner. The increase in gas production and IVOMD observed at 10% inclusion suggests that incorporation of *S. plumosum* in diets improved diet utilization by ruminal microorganisms [55]. The increased gas production and IVOMD at 10% inclusion indicate that 10% inclusion of *S. plumosum* in diets supported efficient ruminal fermentation, which is consistent with enhanced microbial utilization of fermentable substrates [56]. This response may be attributed to a favorable combination of fermentable nutrients supplied by the diet at 10% *S. plumosum* inclusion, which supported ruminal microbial activity and enhanced degradation of dietary organic matter, while the relatively low CT concentration was unlikely to substantially inhibit fermentation [42,56]. Similar responses have been reported for tannin-rich browse species, including *Leucaena leucocephala*, *Vachellia* (*Acacia*) spp. and *Calliandra calothyrsus*, although CT concentrations vary considerably among species, accessions, plant parts, and growing conditions [57,58]. However, the dietary CT concentrations in the present study (0.021–0.032% DM) were substantially lower than those reported for these browse species, which may explain the absence of marked effects on feed intake and methane emissions.

The reduction in gas production and IVOMD at 20% and 30% diets indicates decreased substrate degradation and fermentation efficiency at higher replacement levels [55]. *Seriphium plumosum* fibre degradation may be limited by the high degree of cell wall lignification, which reduces the accessibility of structural carbohydrates to fibrolytic microorganisms [59]. Therefore, the increasing replacement of lucerne hay with *S. plumosum* in the current study may have reached a level at which the higher fiber fraction constrained substrate degradation and ruminal fermentation [60].

Ammonia-N concentrations remained relatively stable across treatments despite increasing replacement of lucerne meal with *S. plumosum* meal. This suggests that ruminal ammonia supply was maintained across treatments and was unlikely to have been a major limiting factor for microbial protein synthesis [61]. Similar findings have been reported in studies evaluating tannin-containing diets, where dietary tannins did not consistently alter ruminal NH_3_-N concentrations when adequate nitrogen was supplied [62,63]. Therefore, the decline in fermentation characteristics observed at the 20% and 30% replacement levels may have been associated with reduced availability of fermentable substrate rather than insufficient nitrogen availability. This interpretation is consistent with the importance of balancing fermentable carbohydrate and nitrogen availability for optimal ruminal microbial growth [64]. When fermentable carbohydrates become limiting, microbial capture of ammonia declines even when ruminal NH_3_-N concentrations remain acceptable. Similarly, [64,65] concluded that microbial protein synthesis is inhibited more by the availability of fermentable organic matter than by ammonia concentration once minimum nitrogen requirements have been contented. 

Total VFA concentration declined significantly as lucerne meal was increasingly replacing *S. plumosum*, up to the value at 20% *S. plumosum* diet. Similar reductions in total VFA production have been reported when highly digestible forages were replaced with more fibrous browse species, such as, *Vachellia nilotica*, *Leucaena leucocephala* [66]. According to Tedeschi et al. [65], differences in cell wall composition and the availability of fermentable carbohydrates are among the main aspects defining fermentation responses to certain forage resources.

The reduction in acetate concentration with increasing *S. plumosum* inclusion levels may reflect reduced fermentation of structural carbohydrates and altered fibrolytic activity within the rumen. Similar reductions in acetate production have been associated with depressed fiber digestion in the presence of plant polyphenols, including tannins, which can inhibit fibrolytic microorganisms and their enzymatic activity [65,67]. Acetate is primarily produced during fibre degradation, and reductions in acetate concentration are usually linked with decreased utilization of structural carbohydrates [65]. The lower acetate concentrations therefore support the observed decline in digestibility and gas production obtained in 20% and 30% diets in the present study.

Propionate concentration declined with increasing *S. plumosum* inclusion, suggesting that replacing lucerne meal with *S. plumosum* meal altered ruminal fermentation patterns. Because dietary NDF and ADF concentrations were similar among treatments, the detected changes in propionate production are unlikely to be explained by differences in fibre content. Instead, they may reflect differences in dietary CP concentration, which varied from 12.6% in the control diet to 13.2% in the 10% inclusion diet and 11.9% in the 20% and 30% inclusion diets. Dietary CP impacts ruminal microbial growth and the availability of ammonia for fermentation, and insufficient nitrogen supply can reduce microbial activity and alter volatile fatty acid production, including propionate [68]. The reduction in acetate concentration with increasing *S. plumosum* inclusion levels indicates reduced fermentation of structural carbohydrates and potentially lower fibrolytic activity in the rumen. Similar reductions in acetate production have been associated with decreased fiber degradation resulting from greater cell-wall lignification and the presence of plant secondary metabolites that can suppress cellulolytic microorganisms [65,67].

### 4.3. Effect of Replacing Lucerne Hay with S. plumosum Meal on Rumen Microbial Composition of Nguni Steers

Although the diets were formulated to evaluate the replacement of lucerne meal with *S. plumosum* meal, maintaining nutritionally balanced diets required adjustments to other ingredients, particularly molasses meal, soybean meal, and feed-grade urea. These changes could have influenced ruminal fermentation by altering the supply of readily fermentable carbohydrates and rumen-degradable nitrogen, which are important substrates for ruminal microbial growth and fermentation [64]. Therefore, differences observed in fermentation characteristics, digestibility, and microbial populations cannot be attributed exclusively to *S. plumosum* inclusion. For example, the higher molasses inclusion in the 10% treatment may have enhanced microbial fermentation by increasing the availability of fermentable carbohydrates, whereas the greater urea inclusion in the 30% treatment likely altered ruminal ammonia availability. Similarly, the variation in dietary crude protein (12.6%, 13.2%, 11.9%, and 11.9%) in respective diets may also have influenced microbial activity.

The selective changes observed in the abundance of the bacterial genera suggest that partial replacement of lucerne with *S. plumosum* influenced specific microbial populations without disrupting the overall rumen bacterial community. This finding agrees with [69] who reported that dietary changes modify the abundance of individual bacterial taxa while the core rumen microbiome remains relatively stable. The reduction in *Erysipelotrichaceae* UCG-009 and the increased abundance of *Ligilactobacillus* with *S. plumosum* inclusion may reflect differential microbial responses to higher fiber and CT concentrations associated with *S. plumosum*. Similar selective responses of rumen microorganisms to tannin-containing forages have been reported [66,67]. This is consistent with the present study, where only modest microbial shifts were observed while nutrient digestibility, ruminal ammonia-N concentration and CH_4_ emissions remained unchanged, suggesting that the observed changes in selected bacterial genera did not substantially alter the measured functional outcomes of rumen fermentation.

## 5. Conclusions

Replacement of lucerne meal with *S. plumosum* meal in diet showed potential as an alternative feed source for Nguni steers by maintaining animal performance while modifying rumen fermentation characteristics and microbial ecosystem. These findings showed that *S. plumosum* in diet improved rumen environment, with microbial community restructuring showing the selection of specific bacterial populations capable of utilizing woody plant species containing recommended CT concentration. The microbial population that was distinguished further supported this adaptive response, showing that dominant and treatment-responsive genera shifted with inclusion of *S. plumosum*. A limitation of the present study is that replacing lucerne required modifications in other dietary ingredients to maintain the diet formulations that meets growing Nguni steers. Future studies should formulate diets with isonitrogenous diet compositions so that the independent effects of *S. plumosum* can be assessed with accuracy. Generally, it can be concluded that *S. plumosum* is a promising locally available forage resource to fill the dry season feed shortage, while supporting productive performance and functional rumen adaptation for ruminants. However, it is recommended that long-term studies under various production systems be conducted to ascertain the present findings.

## Figures and Tables

**Figure 1:**
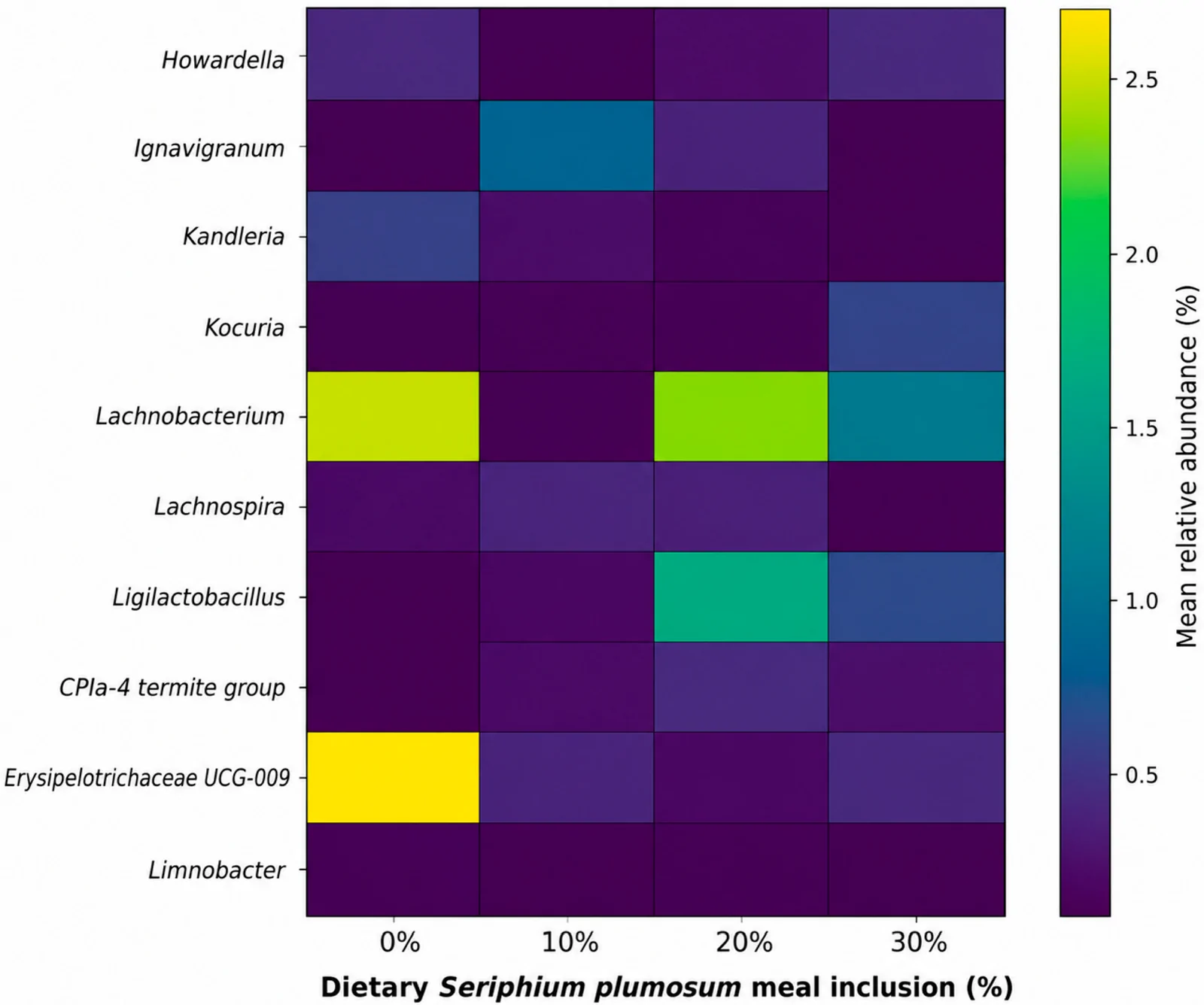
Heatmap illustrating the mean relative abundance (%) of the ten selected bacterial genera in rumen fluid of Nguni steers fed diets containing 0, 10, 20, and 30% *Seriphium plumosum* meal as a replacement for lucerne meal. Warmer colours indicate higher relative abundance, whereas cooler colours indicate lower relative abundance.

**Table 1:** Diet ingredients and nutrient composition.

	Diets ^#^				
	T1_0_	T2_10_	T3_20_	T4_30_	
Feed ingredient (%)					
Lucerne hay	30.0	20.0	10.0	0.0	
Maize meal	55.2	55.2	54.9	55.7	
Molasses meal	8.1	8.6	7.9	5.4	
Soya bean meal	6.0	6.0	6.2	7.2	
Feed-grade urea	0.2	0.6	0.5	1.2	
Cattle salt (NaCl; ≥99% sodium chloride)	0.5	0.5	0.5	0.5	
*S. plumosum*	0	10	20	30	
Nutrient composition *					*S. plumosum*
Dry matter (%)	90.6	91.3	91.0	91.8	87.9
Crude protein (%)	12.6	13.2	11.9	11.9	12.5
Metabolizable Energy (MJ/kg DM)	13.4	13.2	13.2	13.0	-
Ether extract (%)	2.7	2.4	2.3	2.3	2.2
NDF (%)	32.0	32.0	32.1	33.0	66.1
ADF (%)	26.0	26.1	26.2	26.3	37.0
Condensed tannins (%)	0.021	0.024	0.030	0.032	0.23

^#^: Treatments were *Seriphium plumosum* meal levels in the diet at 0 (T1_0_), 10 (T2_10_), 20 (T3_20_) or 30 (T4_30_) %, *: Values are presented as means for each ingredient proportion and each diet.

**Table 2:** Effect of replacing lucerne meal with *S. plumosum* meal in diet on nutrient intake, nutrient digestibility, live weight gain and methane emission of Nguni steers.

		Diets							
Variable	T1 (0%)	T2 (10%)	T3 (20%)	T4 (30%)	SEM	ANOVA P	Linear P	Quadratic P	Cubic P
DMI (kg/d)	5.89	7.82	7.23	8.44	0.75	0.12	0.12	0.19	0.20
CP intake (kg/d)	0.67	0.79	0.76	0.89	0.04	0.08	0.03	0.41	0.62
NDF intake (kg/d)	1.72	2.14	1.97	2.92	0.14	0.10	0.00	0.03	0.71
ME intake (MJ/d)	79.00	103.70	95.50	109.70	8.30	0.41	0.41	0.08	0.62
DM digestibility (%)	76.23	76.43	75.83	75.09	1.08	0.71	0.32	0.48	0.61
CP digestibility	0.70	0.74	0.73	0.74	0.02	0.09	0.18	0.66	0.77
NDF digestibility	0.75	0.71	0.66	0.70	0.03	0.08	0.06	0.03	0.64
ME digestibility (MJ/d)	76.23	76.43	75.83	75.09	1.08	0.71	0.32	0.48	0.61
IBW (kg)	286.71	295.71	299.14	280.71	7.00	0.25	0.64	0.06	0.60
FBW (kg)	337.57	355.29	338.71	351.29	12.84	0.62	0.63	0.82	0.23
BWG (kg)	50.86	59.57	39.57	70.57	11.96	0.50	0.55	0.45	0.23
ADG (kg/day)	0.737	0.863	0.573	1.023	0.210	0.50	0.55	0.45	0.23
Methane emission (ppm)	111.26	99.80	97.89	107.59	7.79	0.62	0.72	0.19	0.95

Mean values of nutrient intake, digestibility coefficients, live weight gain, and methane emissions of Nguni steers fed diets containing 0%, 10%, 20%, and 30% *Seriphium plumosum* meal inclusions are presented in Table 2. SEM = standard error of the mean; DMI = dry matter intake; CP = crude protein; NDF = neutral detergent fibre; ME = Energy intake; DM digestibility = dry matter digestibility; IBW = initial body weight; FBW = final body weight; BWG = body weight gain; ADG = average daily gain. Statistical significance was declared at *p* < 0.05.

**Table 3:** Effect of replacing lucerne hay with increasing levels of *S. plumosum* on in vitro gas production, organic matter degradability, ammonia nitrogen concentration, and volatile fatty acid profiles.

	Diets								
Variable	T1 (0%)	T2 (10%)	T3 (20%)	T4 (30%)	SEM	ANOVA P	Linear P	Quadratic P	Cubic P
Gas production (3 h)	3.32 ^b^	7.15 ^a^	6.92 ^a^	6.38 ^a^	0.38	<0.01	<0.01	0.07	0.39
Gas production (6 h)	4.01 ^c^	7.73 ^a^	7.55 ^a^	5.90 ^b^	0.16	<0001	<0.01	<0.01	0.03
Gas production (12 h)	8.21 ^c^	27.06 ^a^	23.25 ^b^	18.57 ^c^	0.54	<0.01	<0.01	<0.01	<0.01
Gas production (24 h)	8.10 ^c^	23.74 ^a^	21.84 ^ab^	19.81 ^b^	0.64	<0.01	<0.01	<0.01	<0.01
Gas production (48 h)	5.18 ^b^	11.41 ^a^	10.80 ^a^	10.62 ^a^	0.29	<0.01	<0.01	<0.01	<0.01
Cum gas production (48 h)	28.81 ^c^	77.09 ^a^	70.36 ^b^	61.29 ^c^	1.39	<0.01	<0.01	<0.01	<0.01
IVOMD (%)	48.93 ^c^	75.32 ^a^	67.62 ^b^	61.36 ^d^	1.40	<0.01	<0.01	<0.01	<0.01
NH_3_-N (mg NH_3_-N/100 mL)	33.68 ^a^	31.69 ^b^	33.13 ^ab^	33.67 ^a^	1.43	<0.02	<0.01	<0.04	<0.18
Acetic acid mmol/L	63.72 ^a^	59.95 ^a^	50.21 ^c^	56.96 ^b^	2.15	<0.01	0.013	<0.01	<0.01
Propionic acid	40.57 ^a^	36.44 ^b^	25.55 ^c^	34.08 ^b^	2.34	<0.01	<0.01	<0.01	<0.01
Isobutyric acid	6.81 ^a^	6.40 ^a^	5.70 ^b^	6.18 ^ab^	0.20	<0.01	<0.56	<0.042	<0.012
Butyric acid	53.91 ^a^	45.70 ^b^	30.66 ^c^	41.27 ^b^	3.58	<0.001	<0.62	<0.01	<0.01
Iso-valeric acid	12.75	11.70	11.19	11.27	0.39	<0.07	<0.80	<0.07	<0.83
Valeric acid	11.59 ^a^	10.29 ^ab^	9.03 ^b^	9.34 ^b^	0.50	<0.01	<0.86	<0.72	<0.66
Total VFA	189.36 ^a^	170.48 ^b^	132.35 ^c^	159.10 ^b^	1.02	<0.01	<0.01	<0.01	<0.01
Acetate: Propionate ratio	1.57 ^c^	1.65 ^b^	1.96 ^a^	1.67 ^b^	0.01	<0.01	0.26	<0.01	<0.00

Standard error of means = SEM. Linear, quadratic, and cubic effects represent polynomial contrasts for increasing levels of *S. plumosum* inclusion. Means within a row with different superscripts (^a–d^) differ significantly (*p* < 0.05). Volatile fatty acids values were interpreted as concentrations in mmol/L based on the analytical output format provided, rather than mol/100 mol. Estimated total volatile fatty acids (TVFA) were calculated as the sum of measured individual VFAs (acetic, propionic, Isobutyric, butyric, iso-valeric and valeric acids). Acetate-to-propionate ratio =A:P=. Cumulative gas production (Cum gas production). Ammonia-Nitrogen =NH_3_-N=, In vitro organic matter degradability = IVOMD.

## Data Availability

The data presented in this study is available upon request from the corresponding author.
